# Aging With Intellectual and Developmental Disabilities: When Dementia Is Diagnosed or Suspected

**DOI:** 10.1093/geroni/igab046.1675

**Published:** 2021-12-17

**Authors:** Phillip Clark, Kelly Munly

## Abstract

Individuals with lifelong intellectual and developmental disabilities (IDD) have unique needs associated with aging that pose challenges for them and their families. In particular, an increased likelihood for early onset Alzheimer’s disease is a major concern that can place individuals at risk for a host of biomedical, psychological, and social challenges. Faced with providers not trained in how to properly screen for, diagnose, and treat conditions, individuals and families are often left with inadequate care, services, and support. To address these concerns, education for professionals is essential in providing accurate information based on clinical best practices. This symposium presents an innovative and interprofessional model developed by a partnership of geriatrics and IDD educational and service organizations based on Project ECHO (Extension for Community Healthcare Outcomes) methodology. A virtual community is created in which participants both teach and learn from each other through a combination of didactic and case presentations. The first paper describes the ECHO model, including the development of the hub and spoke structure, recruitment of providers, and collaborative and multidisciplinary process of curriculum development. The second paper explores educational experiences of participating spoke agencies in the program, including professionals’ and clients’ outcomes. The third paper presents the implications of creating a foundation based on interprofessional education and networking principles to bridge the gap between health and social care disciplines and parallel service systems. The final paper provides recommendations and implications for developing and refining methods to address the need for provider education in this rapidly expanding field.

